# Scrapping the Trained Nurse: The Only Alternative

**Published:** 1920-04-10

**Authors:** Gladys M. E. Leigh


### THE EDITOR'S LETTER-BOX.

**Correspondence on all subjects is invited, but we cannot in any way be responsible for the opinions expressed by our correspondents, who must give their name and address as a guarantee of good faith, but not necessarily for publication. Correspondents are reminded that brevity of style and conciseness of statement greatly facilitate early insertion.**

### Scrapping the Trained Nurse: The Only Alternative.

*To the Editor of* THE HOSPITAL.

SIR,—I am in complete agreement with your correspondent Ierne on two points, viz. :—

(*a*) The attitude of either man or woman who will never admit he (or she) can make a mistake is not of vital importance.

(*b*) That controversy in the nursing press is inimical to the interests of the trained nurse, whereas concerted action between the heads of the profession and the rank-and-file may accomplish practical results.

If the trained nurse is not to be ground between the mill-stones of red-tape and philanthropy, she must act *now—at once*—and with decision. The first step is to convince the Ministry of Health and the general public that the nursing services consist of women who are educated, organised, efficient, and willing, and able, to protest by constitutional methods against a decision they consider inimical not only to their own interests, but to those of the sick poor.

There is nothing, that I am aware, in the constitution of the College of Nursing to prevent the chairman convoking a special meeting of the Council to consider the aspect of the curriculum laid down for health visitors in its relation to the interests of the trained nurse. If the Council is not satisfied with the concessions granted to members of the profession, let a referendum be taken of the opinion of the rank-and-file of the College members.

I can see no impediment against the Local Centres issuing ballot-papers: Are you satisfied with the concessions granted to trained women who desire to qualify as health visitors? Are you willing to accept service under existing conditions? I venture to predict the answers would be in the negative. The Council would then be in a position to request Dr. Addison to receive a deputation to lay before him the opinion of a considerable section of the nursing services with regard to the conditions of training for health visitors; moreover, this deputation might be authorised to ask that delegates appointed by the Minister of Health might meet an equal number of delegates appointed by the various recognised nursing organisations in the hope that a settlement agreeable to both might be reached.

If this conference breaks down, let the matter be referred to arbitration. Concerted action, as I suggest, could not fail to enlist the entire sympathy of all professional associations, and would have a far-reaching and beneficial reaction on the social and economic status of the nursing services.—Yours faithfully,

GLADYS M. E. LEIGH.

